# Tumor microenvironment in triple-negative breast cancer: the correlation of tumor-associated macrophages and tumor-infiltrating lymphocytes

**DOI:** 10.1007/s12094-021-02652-3

**Published:** 2021-06-05

**Authors:** H. Kuroda, T. Jamiyan, R. Yamaguchi, A. Kakumoto, A. Abe, O. Harada, A. Masunaga

**Affiliations:** 1 Department of Diagnostic Pathology, Tokyo Women’s Medical University, Medical Center East, 2-1-10 Nishiogu, Arakawa-ku, Tokyo, 116-8567 Japan; 2 Department of Diagnostic Pathology, Dokkyo Medical University, Mibu, Japan; 3 Department of Pathology and Forensic Medicine, Mongolian National University of Medical Sciences, Ulaanbaatar, Mongolia; 4 Department of Pathology & Laboratory Medicine, Kurume University Medical Center, Kurume, Japan; 5 Department of Diagnostic Pathology, Nasu Red Cross Hospital, Otawara, Japan; 6 Breast Center, Dokkyo Medical University, Mibu, Japan; 7 Breast Center, Showa University, Tokyo, Japan

**Keywords:** Triple-negative breast cancer, Tumor microenvironment, Tumor-infiltrated immune cells, Tumor-associated macrophages, Tumor-infiltrating lymphocytes

## Abstract

**Purpose:**

Immune cells such as cytotoxic T cells, helper T cells, B cells or tumor-associated macrophages (TAMs) contribute to the anti-tumor response or pro-tumorigenic effect in triple negative breast cancer (TNBC). The interrelation of TAMs, T and B tumor-infiltrating lymphocytes (TILs) in TNBC has not been fully elucidated.

**Methods:**

We evaluated the association of tumor-associated macrophages, T and B TILs in TNBC.

**Results:**

TNBCs with a high CD68+, CD163+ TAMs and low CD4+, CD8+, CD20+ TILs had a significantly shorter relapse-free survival (RFS) and overall survival (OS) than those with low CD68+, CD163+ TAMs and high CD4+, CD8+, CD20+ TILs. TNBCs with high CD68+ TAMs/low CD8+ TILs showed a significantly shorter RFS and OS and a significantly poorer prognosis than those with high CD68+ TAMs/high CD8+ TILs, low CD68+ TAMs/high CD8+ TILs, and low CD68+/low CD8+. TNBCs with high CD163+ TAMs/low CD8+, low CD20 + TILs showed a significantly shorter RFS and OS and a significantly poorer prognosis than those with high CD163+ TAMs/high CD8+ TILs and high CD163+ TAMs /high CD20+ TILs.

**Conclusions:**

Our study suggests that TAMs further create an optimal tumor microenvironment (TME) for growth and invasion of cancer cells when evasion of immunoreactions due to T and B TILs occurs. In TNBCs, all these events combine to affect prognosis. The process of TME is highly complex in TNBCs and for an improved understanding, larger validation studies are necessary to confirm these findings.

## Introduction

Cancers historically described as medullary carcinoma, or carcinoma with medullary features, were previously recognized as a specific, special type of well-circumscribed breast cancer with a prominent tumor-infiltrating lymphocyte (TIL) and macrophage infiltrate, and associated with better prognosis than other stage-matched high-grade cancers. However, “carcinoma with medullary features” has suffered from poor interobserver reproducibility and overlapping features with Triple-negative breast cancer (TNBC). TNBC is characterized by a lack of expression of the estrogen receptor (ER) and progesterone receptor (PgR), and absence of human epidermal growth factor receptor 2 (HER2) protein overexpression; this type is known to have a poor prognosis and to recruit TILs and tumor-associated macrophages (TAMs). Usually, hormone therapy and drugs that target HER2 are not helpful in TNBC, so chemotherapy is the main systemic treatment option. In addition, there is currently no consideration of these immune cells in the therapeutic approach. A more recent discovery of the prognostic importance of TILs in high-grade breast cancers appears to explain the good prognosis of these cancers [1–3]. The latest WHO classification proposed considering carcinomas with a medullary pattern as representing one end of the spectrum of TIL-rich invasive breast carcinoma of no special type (IBC-NSTs), rather than a distinct morphological subtype, and to use the term “IBC-NST with medullary pattern” [4].

According to these series of theories, we previously reported that CD20+ TILs may support an increase in CD4+ and CD8+ TILs, altering the anti-tumor response and resulting in a positive prognosis in TNBC [5–7]. Further, although the prognostic correlation with macrophages has been widely reported in breast cancer, there is no consensus on the prognostic impact [8–12]. TAMs have recently been reported as an important factor in tumor growth and cancer progression. Recently, two processes have been proposed for TAMs activation: Classically-activated type 1 (M1-like) macrophages and alternatively-activated type 2 (M2-like) macrophages. M1-like macrophages, characterized by CD68 expression, produce free radicals that can lead to DNA damage with the potential to contribute to tumoricidal activity [13]. In contrast, M2-like macrophages, characterized by both CD68 and CD163 expression, are considered to promote tumor growth and metastasis by releasing chemokines, which are inflammatory growth factors [14, 15].

CD68+ and CD163+ are the most commonly reported when identifying TAM. In addition, we previously reported that infiltration of CD163+ TAMs, rather than CD68+, was associated with poor prognosis in TNBC patients by multivariate analysis [16]. To date, only a few studies have investigated TILs and macrophages in combination [17–19]. Recent discoveries about the immune system have drastically changed conventional assumptions regarding the role of macrophages and lymphocytes in anti-tumor activity [6, 20–23]. TAMs cooperate with T and B TILs based on the release of chemokines, cytokines with reactive radicals among other proteins. Recently, tumor microenvironment, such as TAMs and TILs have been considered important prognostic factors in cancer. However, the interrelation of TAMs, T and B TILs in TNBC has not been fully elucidated. The purpose of the present study was to evaluate the CD68+ and CD163+ TAMs, CD4+, CD8+ T TILs and CD20+ B TILs in TNBC and examine their clinicopathological features and correlations.

## Methods

### Patients and tissue specimens

A total 107 cases of TNBC who had operations from 2006 to 2018 in Dokkyo Medical University hospital were used in the present study. The explored clinicopathological parameters included age, tumor size, histologic grade, histology, lymph node status, mib-1 index, unstained TILs, and follow-up data. This study was conducted according to the Declaration of Helsinki and after approval by the ethics committee of Dokkyo Medical University (No.28009).

Immunohistochemistry (IHC) was performed on formalin-fixed and paraffin-embedded sections using an antibody to ER (clone SP1, Novocastra (Leica), prediluted, nuclear), PgR (clone 1E2, Novocastra (Leica), prediluted, nuclear), HER2 (clone 4B5, Roche (VENTANA), prediluted, membranous), CD4 (CD4, clone 1F6, Novocastra (Leica), 1:40), CD8 (CD8, clone 4B11, Novocastra (Leica), prediluted), CD20 (CD20, clone L26, Nichirei), CD68 (CD68, clone PG-M1, Dako (Agilent), 1:50), and CD163 (CD163, clone 10D6, Novocastra (Leica), 1:50), ki67 (mib-1, clone K-2, Novocastra (Leica)) according to the manufacturer’s instructions. For every IHC staining, tonsil specimens were used as positive and negative controls.

The percentages of nuclei stained for ER and PgR expression were analyzed using the American Society of Clinical Oncology/College of American Pathologists (ASCO/CAP) guidelines of a threshold of 1% [24]. HER2 expression was assessed according to the guidelines determined by ASCO/CAP [25]. Analysis of unstained TILs was performed on hematoxylin and eosin-stained sections according to the criteria proposed by the International Immuno-Oncology Biomarkers Working Group [26]. Unstained TILs were defined as lymphocytes located within the stroma and stratified as high (≥ 30%) and low (< 30%) [27]. The mib-1 index was derived from the sum total of the percentages of different staining intensities, and threshold of the set at 20% [28].

**Fig. 1:**
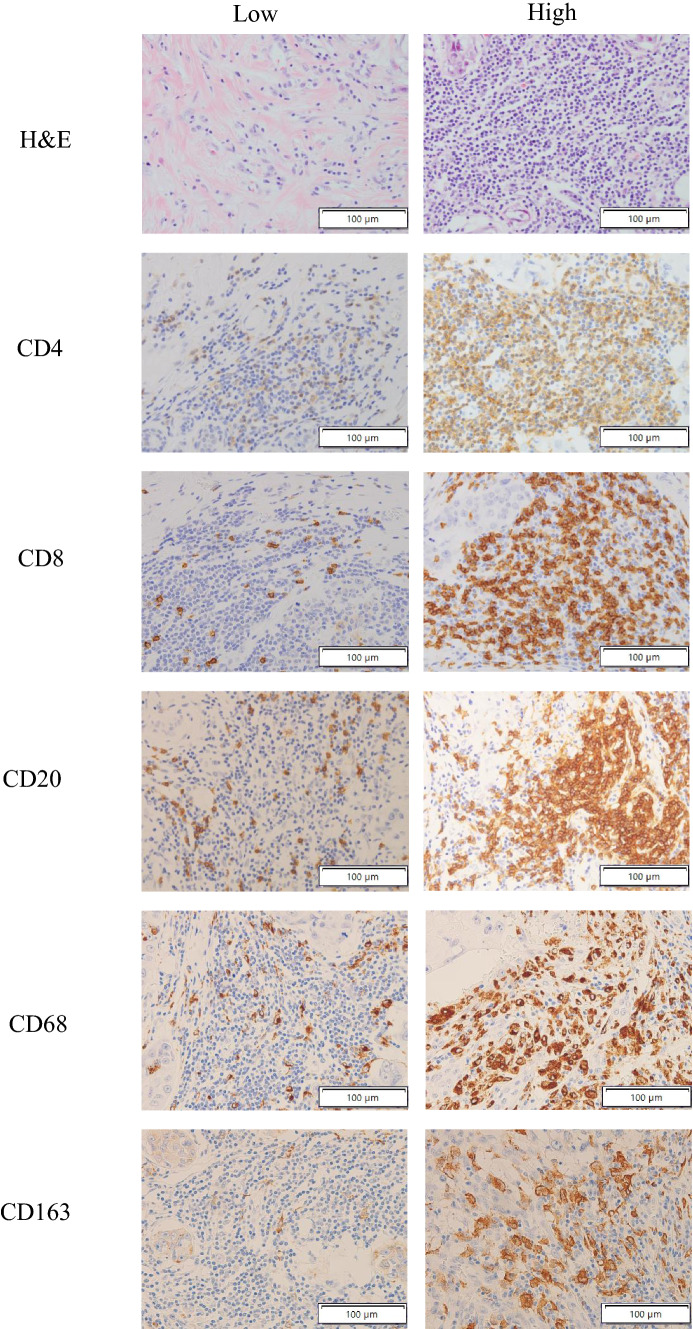
Representative hematoxylin and eosin and immunohistochemistry images showing tumor microenvironments with lower and higher densities. At 400× magnification

The density of tumor-infiltrating immune cell subsets in the tumor stroma of TNBC was quantified as total counts of CD4, CD8, CD20, CD68, and CD163-positive cells per high power field by manual inspection of stained sections with five areas of high staining intensity. Each specimen was screened at low magnification (×100), and the greatest number of positively stained cells (hot spot area) was selected for the subsequent analysis. The mean tumor-infiltrating immune cell counts in these areas for each case were evaluated. Infiltrating immune cells, as identified by the different markers, and the number of positive cells were divided into lower and higher groups (Fig. 1) based on cut-off points according to the median. As a result, the cut-off for CD4 was 104, CD8 was 81, CD20 was 60, CD68 was 26.2, and CD163 was 26.6. All slides were estimated by two pathologists (HK and TJ) who had no access to the clinical data.

### Statistical analysis

All statistical analysis was performed using SPSS 26.0 (IBM Corporation, Armonk, NY, USA). A *p* value of < 0.05 was regarded as significant and all statistical tests were two-sided. Correlation analyses between TAMs or TILs and clinicopathological parameters were done using Pearson’s chi-square test. The correlation of TAMs and TILs with relapse-free survival (RFS) and overall survival (OS) was analyzed by Kaplan–Meier analysis. Significance was evaluated using the log-rank test. Cox proportional hazard models were used to estimate hazard ratios for death from breast cancer according to the correlation of TAMs and TILs in both univariate and multivariate analyses.

## Results

The clinicopathological parameters of the patients are summarized in Tables 1 and 2. The density of CD68+ TAMs/CD8+ TILs was significantly associated with histology (*p* = 0.020) and the mib-1 index (*p* = 0.043). Moreover, the densities of CD68+ TAMs/CD8+ TILs and CD68+ TAMs/CD20+ TILs were significantly related to expression of unstained TILs (*p* = 0.022, *p* < 0.001). Additionally, histological grade was significantly associated with CD163+ TAMs/CD4+ TILs or CD8+ TILs or CD20+ TILs (*p* < 0.001, *p* < 0.001, and *p* = 0.001; respectively). The CD163+ TAMs/CD4+ TILs (*p* = 0.038) and CD163+ TAMs/CD8+ TILs (*p* = 0.020) were correlated with histology. Furthermore, CD163+ TAMs/CD8+ TILs and CD163+ TAMs/CD20+ TILs were significantly related to expression of unstained TILs (*p* = 0.022, *p* < 0.001). However, there was no significant difference found in other clinicopathological parameters.

**Table 1:** Associations with clinicopathological parameters and TAMs versus TILs status

Clinicopathological parameter	CD68/CD4					CD68/CD8					CD68/CD20				
	High/low	High/high	Low/high	Low/low	*p* value	High/low	High/high	Low/high	Low/low	*p* value	High/low	High/high	Low/high	Low/low	*p* value
Age at diagnosis (years)					0.462					0.571					0.618
< 60	11	15	14	12		13	14	14	11		13	13	14	12	
≥ 60	17	10	13	15		12	14	11	18		18	9	17	11	
Tumor size (cm)					0.124					0.727					0.131
≤ 2	13	15	21	17		13	18	16	19		15	13	24	14	
> 2	15	10	6	10		12	10	9	10		16	9	7	9	
Histological grade					0.082					0.089					0.224
I, II	6	5	7	13		5	6	5	15		7	4	10	10	
III	22	20	20	14		16	26	16	18		24	18	21	13	
Histology					0.080					*0.020*					0.498
IBC-NST	19	19	21	18		17	21	20	19		20	18	21	18	
IBC-NST with medullary pattern	2	6	3	0		1	6	4	0		4	4	3	0	
Ca with apocrine differentiation	4	0	3	3		3	0	1	6		4	0	4	2	
Metaplastic carcinoma	2	0	0	4		3	1	0	2		2	0	2	2	
ILC	1	0	0	1		0	0	0	2		1	0	1	0	
IMP	0	0	0	1		1	0	0	0		0	0	0	1	
Lymph node status					0.578					0.532					0.729
Absent	13	15	18	19		9	19	14	23		17	11	21	16	
Present	11	6	7	6		8	9	6	7		9	8	8	5	
N/A	4	4	2	2		4	4	1	3		5	3	2	2	
Mib-1 index					0.065					*0.043*					0.068
< 20%	2	3	5	9		2	3	3	11		3	2	6	8	
≥ 20%	26	22	22	18		19	29	18	22		28	20	25	15	
Unstained TILs					0.198					*0.022*					< *0.001*
Low (≤ 30%)	17	9	15	17		16	12	9	21		22	4	10	22	
High (> 30%)	11	16	12	10		9	16	16	8		9	18	21	1	

*IBC-NST* invasive breast carcinoma of no special type, *Ca* carcinoma, *ILC* invasive lobular carcinoma, *IMP* invasive micropapillary carcinoma, *N/A* not applicable, *TILs* tumor-infiltrating lymphocytes

*χ*^2^ test. Italic type indicates a statistically significant difference (*p* < 0.05)

**Table 2:** Associations with clinicopathological parameters and TAMs versus TILs status

Clinicopathological parameter	CD163/CD4					CD163/CD8					CD163/CD20				
	High/low	High/high	Low/high	Low/low	*p* value	High/low	High/high	Low/high	Low/low	*p* value	High/low	High/high	Low/high	Low/low	*p* value
Age at diagnosis (years)					0.511					0.571					0.924
< 60	12	15	14	11		13	14	14	11		13	14	13	12	
≥ 60	15	11	12	17		12	14	11	18		14	12	14	15	
Tumor size (cm)					0.168					0.727					0.213
≤ 2	12	19	17	18		13	18	16	19		15	16	21	14	
> 2	15	7	9	10		12	10	9	10		12	10	6	13	
Histological grade					< *0.001*					< *0.001*					*0.001*
I, II	2	4	8	17		3	3	8	17		4	2	12	13	
III	25	22	18	11		22	25	17	12		23	24	15	14	
Histology					*0.038*					*0.020*					0.717
IBC-NST	19	19	21	18		17	21	20	19		19	19	20	19	
IBC-NST with medullary pattern	1	6	3	1		1	6	4	0		2	5	2	2	
Ca with apocrine differentiation	2	1	2	5		3	0	1	6		2	1	3	4	
Metaplastic carcinoma	0	0	0	2		3	1	0	2		3	1	1	1	
ILC	0	0	0	2		0	0	0	2		0	0	1	1	
IMP	1	0	0	0		1	0	0	0		1	0	0	0	
Lymph node status					0.357					0.569					0.490
Absent	18	15	12	20		12	15	18	20		13	14	18	20	
Present	6	7	12	5		9	10	5	6		10	9	7	4	
N/A	2	4	3	3		4	3	2	3		4	3	2	3	
Mib-1 index					0.324					0.124					0.168
< 20%	4	2	6	7		4	2	4	9		5	1	7	6	
≥ 20%	23	24	20	21		21	26	21	20		22	25	20	21	
Unstained TILs					0.223					*0.022*					< *0.001*
Low (≤ 30%)	18	10	14	16		16	12	9	21		22	6	8	22	
High (> 30%)	9	16	12	12		9	16	16	8		5	20	19	5	

*IBC-NST* invasive breast carcinoma of no special type, *Ca* carcinoma, *ILC* invasive lobular carcinoma, *IMP* invasive micropapillary carcinoma, *N/A* not applicable, *TILs* tumor-infiltrating lymphocytes

*χ*^2^ test. Italic type indicates a statistically significant difference (*p* < 0.05)

**Fig. 2:**
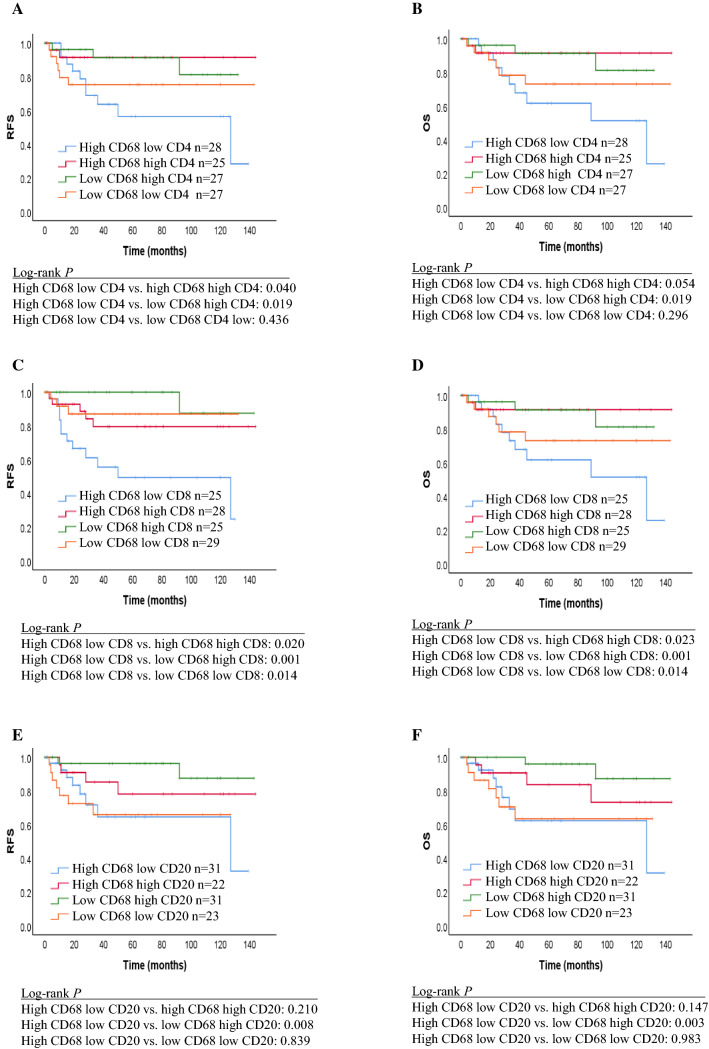
Kaplan–Meier curves showing the relapse-free survival and overall survival of patients with certain densities of CD68+ TAMs versus densities of TILs. **a**, **b** CD68+ TAMs/CD4+ TILs. **c**, **d** CD68+ TAMs/CD8+ TILs. **e**, **f** CD68+ TAMs/CD20+ TILs. *TILs* tumor-infiltrating lymphocytes, *TAMs* tumor-associated macrophages

**Fig. 3:**
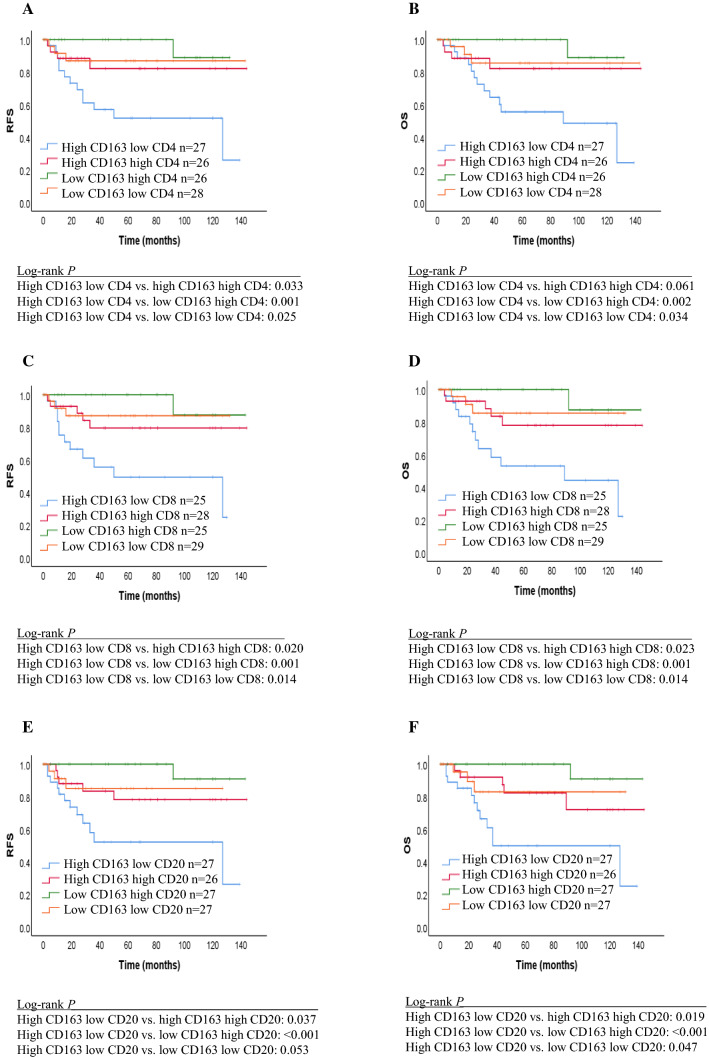
Kaplan–Meier curves showing the relapse-free survival and overall survival of patients with certain densities of CD163+ TAMs versus densities of TILs. **a**, **b** CD163+ TAMs/CD4+ TILs. **c**, **d** CD163+ TAMs/CD8+ TILs. **e**, **f** CD163+ TAMs/CD20+ TILs. *TILs* tumor-infiltrating lymphocytes, *TAMs* tumor-associated macrophages

RFS and OS rates for all groups are shown by Kaplan–Meier curves and differences were analyzed by the log-rank test (Figs. 2, 3). Patients with a high CD68+ TAMs/low CD4+ TILs phenotype had a statistically significant shorter RFS and OS compared with patients with high CD68+ TAMs/high CD4+ TILs (RFS: *p* = 0.040) and low CD68+ TAMs/high CD4+ TILs (RFS: *p* = 0.019, OS: *p* = 0.019). There was no significant difference found among high CD68+ TAMs/low CD4+ TILs, high CD68+ TAMs/high CD4+ TILs and low CD68+ TAMs/low CD4+ TILs (RFS: *p* = 0.436, OS: *p* = 0.296). Patients with a high CD68+ TAMs/low CD8+ TILs phenotype had a statistically significant shorter RFS and OS compared with those with high CD68+ /high CD8+ TILs (RFS: *p* = 0.020, OS: *p* = 0.023), low CD68+ TAMs/high CD8+ TILs (RFS: *p* = 0.001, OS: *p* = 0.001), and low CD68+ TAMs/low CD8+ TILs (RFS: *p* = 0.014, OS: *p* = 0.014). Patients with a high CD68+ TAMs/low CD20+ TILs phenotype had a statistically significant shorter RFS and OS compared with patients with low CD68+ TAMs/high CD20+ TILs (RFS: *p* = 0.008, OS: *p* = 0.003). No significant difference was found among high CD68+ TAMs/low CD20+ TILs, high CD68+ TAMs/high CD20+ TILs (RFS: *p* = 0.210, OS: *p* = 0.147) and low CD68+ TAMs/low CD20+ TILs (RFS: *p* = 0.839, OS: *p* = 0.983).

For TAM2s/TILs, patients with a high CD163+ TAMs/low CD4+ TILs phenotype had a statistically significant shorter RFS and OS compared with those with high CD163+ TAMs/high CD4+ TILs (RFS: *p* = 0.033), low CD163+ TAMs/high CD4+ TILs (RFS: *p* = 0.001, OS: *p* = 0.002) and low CD163+ TAMs/low CD4+ TILs (RFS: *p* = 0.025, OS: *p* = 0.034). No significant difference was observed between high CD163+ TAMs/lowCD4+ TILs and high CD163+ TAMs/high CD4+ TILs (OS: *p* = 0.061) in OS. Patients with a high CD163+ TAMs/low CD8+ TILs phenotype had a statistically significant shorter RFS and OS compared with those with high CD163+ TAMs/high CD8+ TILs (RFS: *p* = 0.020, OS: *p* = 0.023), low CD163+ TAMs/high CD8+ TILs (RFS: *p* = 0.001, OS: *p* = 0.001) and low CD163+ TAMs/low CD8+ TILs (RFS: *p* = 0.014, OS: *p* = 0.014). Patients with a high CD163+ TAMs/low CD20+ TILs phenotype had a statistically significant shorter RFS and OS compared with those with high CD163+ TAMs/high CD20+ TILs (RFS: *p* = 0.037, OS: *p* = 0.019), low CD163+ TAMs/high CD20+ TILs (RFS: *p* < 0.001, OS: *p* < 0.001) and low CD163+ TAMs/low CD20+ TILs (OS: *p* = 0.047). There was no significant difference between high CD163+ TAMs/low CD20+ TILs and low CD163+ TAMs/low CD20+ TILs (RFS: *p* = 0.053) in RFS.

To evaluate the prognostic implications of CD68+ and CD163+ TAMs in relation to infiltrating CD4+, CD8+, CD20+ TILs, we compared the prognostic value of groups of CD68+ or CD163+ (high or low) to those of CD4+, CD8+ and, CD20+ (high or low) (Table 3 and 4). In the multivariate assessment, patients with a high CD68+ TAMs/low CD8+ TILs phenotype had a statistically significantly poorer prognosis compared to patients with high CD68+ TAMs/high CD8+ TILs (RFS: hazard ratio (HR) 0.334, 95% CI 0.117–0.952, *p* = 0.040, OS: HR 0.330, 95% CI 0.116–0.940, *p* = 0.038), low CD68+ TAMs/high CD8+ TILs (RFS: HR 0.073, 95% CI 0.009–0.569, *p* = 0.013, OS: HR 0.074, 95% CI 0.009–0.576, *p* = 0.013), and low CD68+ TAMs/low CD8+ TILs (RFS: HR 0.214, 95% CI 0.055–0.827, *p* = 0.025, OS: HR 0.219, 95% CI 0.056–0.861, *p* = 0.030).

**Table 3:** Univariate and multivariate analysis of TAMs/TILs related to RFS and OS of TNBC patients in the cohort

Immune markers	*n*	Univariate						Multivariate					
		RFS			OS			RFS			OS		
		HR	95% CI	*p* value	HR	95% CI	*p* value	HR	95% CI	*p* value	HR	95% CI	*p* value
CD68/CD4													
High/low	28		Reference			Reference							
High/high	25	0.233	0.051–1.064	0.06	0.247	0.054–1.130	0.071						
Low/high	27	0.263	0.072–0.958	*0.043*	0.253	0.069–0.921	*0.037*	0.152	0.018–1.304	0.086	0.165	0.020–1.363	0.094
Low/low	27	0.657	0.238–1.814	0.418	0.586	0.213–1.614	0.301						
CD68/CD8													
High/low	25		Reference			Reference							
High/high	28	0.318	0.112–0.904	*0.032*	0.323	0.113–0.918	*0.034*	0.334	0.117–0.952	*0.04*	0.33	0.116–0.940	*0.038*
Low/high	25	0.074	0.010–0.570	*0.012*	0.075	0.010–0.578	*0.013*	0.073	0.009–0.569	*0.013*	0.074	0.009–0.576	*0.013*
Low/low	29	0.232	0.065–0.823	*0.024*	0.231	0.065–0.819	*0.023*	0.214	0.055–0.827	*0.025*	0.219	0.056–0.861	*0.03*
CD68/CD20													
High/low	31		Reference			Reference							
High/high	22	0.478	0.143–1.599	0.231	0.423	0.126–1.424	0.165						
Low/high	31	0.17	0.036–0.812	*0.026*	0.142	0.030–0.680	*0.015*	0.568	0.044–7.250	0.663	0.536	0.038–7.559	0.644
Low/low	23	1.061	0.383–2.940	0.909	0.917	0.330–2.546	0.868						

*TAMs* tumor-associated macrophages, *TILs* tumor-infiltrating lymphocytes, *RFS* relapse-free survival, *OS* overall survival, *TNBC* triple-negative breast cancer, *HR* hazard ratio, *CI* confidence interval

Italic type indicates a statistically significant difference (*p* < 0.05)

**Table 4:** Univariate and multivariate analysis of TAMs/TILs related to RFS and OS of TNBC patients in the cohort

Immune markers	*n*	Univariate						Multivariate					
		RFS			OS			RFS			OS		
		HR	95% CI	*p* value	HR	95% CI	*p* value	HR	95% CI	*p* value	HR	95% CI	*p* value
CD163/CD4													
High/low	27		Reference			Reference							
High/high	26	0.323	0.105–0.993	*0.049*	0.363	0.118–1.114	0.076	0.356	0.113–1.127	0.079			
Low/high	26	0.077	0.010–0.593	*0.014*	0.081	0.011–0.622	*0.016*	0.15	0.010–2.244	0.169	0.191	0.013–2.781	0.226
Low/low	28	0.268	0.076–0.940	*0.04*	0.281	0.080–0.987	*0.048*	0.527	0.057–4.860	0.572	0.69	0.075–6.302	0.742
CD163/CD8													
High/low	25		Reference			Reference							
High/high	28	0.318	0.112–0.904	*0.032*	0.323	0.113–0.918	*0.034*	0.339	0.118–0.969	*0.044*	0.344	0.121–0.984	*0.047*
Low/high	25	0.074	0.010–0.570	*0.012*	0.075	0.010–0.578	*0.013*	0.18	0.013–2.431	0.197	0.21	0.016–2.772	0.236
Low/low	29	0.232	0.065–0.823	*0.024*	0.231	0.065–0.819	*0.023*	0.647	0.069–6.033	0.702	0.772	0.085–7.028	0.818
CD163/CD20													
High/low	27		Reference			Reference							
High/high	26	0.346	0.121–0.989	*0.048*	0.311	0.109–0.890	*0.03*	0.378	0.132–1.086	0.071	0.336	0.117–0.965	*0.043*
Low/high	27	0.064	0.008–0.497	*0.009*	0.059	0.008–0.455	*0.007*	0.237	0.017–3.371	0.288	0.222	0.016–3.153	0.266
Low/low	27	0.308	0.087–1.093	0.068	0.299	0.084–1.062	0.062						

*TAMs* tumor-associated macrophages, *TILs* tumor-infiltrating lymphocytes, *RFS* relapse-free survival, *OS* overall survival, *TNBC* triple-negative breast cancer, *HR* hazard ratio, *CI* confidence interval

Italic type indicates a statistically significant difference (*p* < 0.05)

Patients with a high CD163+ TAMs/low CD8+ TILs phenotype had a statistically significant poorer prognosis compared with patients with high CD163+ TAMs/high CD8+ TILs (RFS: HR 0.339, 95% CI 0.118–0.969, *p* = 0.044, OS: HR 0.344, 95% CI 0.121–0.984, *p* = 0.047). Interestingly, patients with a high CD163+ TAMs/low CD8+ TILs phenotype had a statistically significant shorter OS compared to patients with CD163+ TAMs/high CD8+ TILs (OS: HR 0.336, 95% CI 0.117–0.915, *p* = 0.043). However, no significant difference was observed in RFS between high CD163+ TAMs/low CD8+ TILs and high CD163+ TAMs/high CD8+ TILs (RFS: HR 0.378, 95% CI 0.132–1.086, *p* = 0.071).

## Discussion

Our study revealed that high TAMs (CD68, CD163) with low TILs (CD4, CD8, CD20) correlated significantly with poor prognosis in TNBCs. Further, multivariate analysis also showed that CD68/CD8, CD163/CD8, and CD163/CD20 were associated with prognosis.

**Fig. 4:**
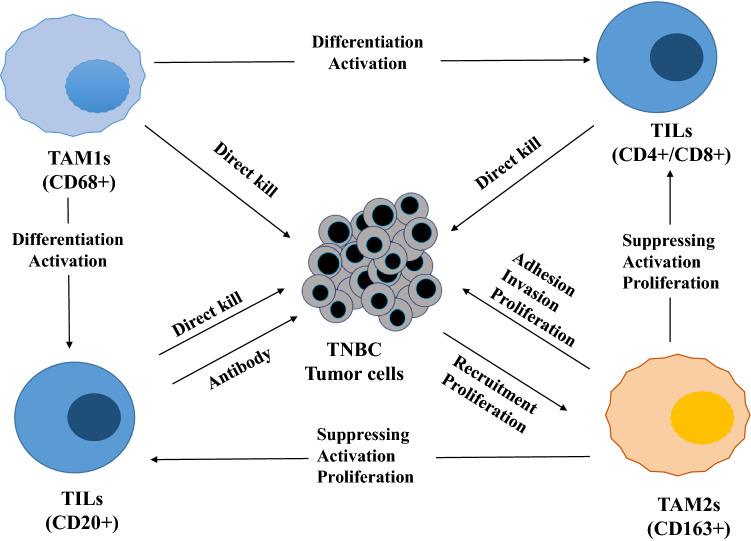
Schema of the interaction of immune cells in the breast cancer tumor microenvironment. CD4+ /CD8+ /CD20+ TILs directly kill the tumor cells. TILs are supported by TAM1s and suppress the tumor cells. In contrast, TAM2s exerts an immunosuppressive function via the inhibition of T cells and B cells, indicating tumorigenic roles. *TILs* tumor-infiltrating lymphocytes, *TAM1s* tumor-associated macrophages-1, *TAM2s* tumor-associated macrophages-2

The first explanation for this result is that immune evasion caused by TAM. TAMs usually play an important role in the human immune system against tumors, and they can induce specific immunity by promoting activation and recruitment of T and B TILs (Fig. 4). They are important to elicit an appropriate immune response [13–15]. B cells T lymphocytes play an essential part in immune defense. Traditionally, these cells are divided into two subtypes, CD4+ T helper cells and CD8+ cytotoxic T cells [29]. The first subtype can help B TILs to induce antibodies to produce immune activity through the recruitment of various immune cells to appropriate sites associated with macrophage response and inflammation. The second type is essential for protection against cytopathogens, among other functions. These immune reactions contribute to pathogen protection, inflammation mitigation, and antibody production. Despite previous reports that suggested a favorable prognosis with early T TILs in TNBCs, successful tumors that progress and become lethal to patients are clearly not eliminated. This immune evasion is at least partly blocked by TAMs, but also involves regulatory T cells, as well as tumor cell immune evasion [30–32]. CD4+ T TILs recruitment in tumors often follows a regulatory T cell (Treg) subset, which leads to severe immunodeficiency and can be produced by TAMs through cytokine expression, as reported in ovarian cancer patients [33]. Similarly, CD4+ T TILs were shown to differentiate into Tregs when co-cultured with TAMs from glioblastoma patients [34]. However, the functional role CD4+ T TILs play in immunological response is complex [35]. CD4+ T TILs with cytotoxic capacity were shown to reject tumor cells and incorporate B TILs release of cytokines to drive cytotoxic immune responses to the tumor cells. The increased expression of CD8+ T TILs was also partly regulated by TAMs and was more pronounced at killing tumors than CD4+ T TILs, for which Treg was more prominent [36, 37].

In this important context, the combination of a high density of TAMs and low infiltration of CD4+, CD8+ T TILs in human breast cancer is indicative of poorer survival [38]. In our study, TNBCs with absent/low infiltration of T and B TILs and a high density of CD68+, CD163+ TAMs had a statistically significant shorter RFS and OS. Similar to our findings, several articles reported that a high density of TAMs was associated with poor prognosis in patients with prostate, urinary bladder, kidney and breast cancer [12, 39–41].

The second explanation is that TAMs can contribute to tumor destruction and influence tumor growth and progression themselves. Previously, we also reported CD163+ TAM2 are correlated in TNBC. TAM receptors are reported to be overexpressed in both solid and hematological malignancies, and high expression of the TAM receptors has been associated with poor patient survival in a variety of cancers. Oncogenic TAM receptor signaling results in increased proliferation, cell survival and metastasis. Actually, our report suggests that CD163+ TAMs may correlate with the poor prognosis of TNBC [16]. However, in this study, even if CD163+ TAM was expressed, the prognosis differed depending on the degree of TIL infiltration. This may be because CD163+ TAM promotes cancer infiltration, while TIL causes an immune response to the cancer, affecting prognosis. Similar results of TAM were obtained with CD68, but were not as significant as with CD163. Some studies of CD68+ TAM involving patients with melanoma, gastric and colorectal cancer reported contrasting findings [42–44].

A possible inference from these opposing results may be the use of a pan-macrophage marker for TAMs. CD68 determines not only TAM2, but also TAM1, which generates free radicals that can induce DNA damage with the potential for tumoricidal activity. Thus, our study suggests that CD163+ TAMs further create an optimal TME for growth and invasion of cancer cells when evasion of immunoreactions by T and B TILs occurs. However, CD68+ TAMs that are thought to be partly derived from TAM1s, when with CD8+ TILs, are suggested to be involved in effective anti-tumor immunity.

## Conclusion

Our study revealed that high TAMs (CD68, CD163) with low TILs (CD4, CD8, CD20) correlated significantly with poor prognosis in TNBCs. Further, multivariate analysis also showed that CD68/CD8, CD163/CD8, and CD163/CD20 were associated with prognosis. Our study suggests that CD163+ TAMs further create an optimal TME for growth and invasion of cancer cells when evasion of immunoreactions by T and B TILs occurs. CD68+ TAMs, which are thought to be partly derived from TAM1s are thought to be involved in effective human anti-tumor immunity. In TNBCs, all these events combine to affect prognosis. The process of TME is highly complex in TNBCs and for an improved understanding, larger validation studies are necessary to confirm these findings.

## Data Availability

The datasets used and/or analyzed during the current study are available from the corresponding author on reasonable request.
